# Bacterial communities associated with the blue dragon nudibranch *Glaucilla marginata* from Phuket, Thailand

**DOI:** 10.7717/peerj.21462

**Published:** 2026-06-19

**Authors:** Sornsiri Phongphattarawat, Nut Songvorawit

**Affiliations:** 1 Marine Microbiology Laboratory, Faculty of Technology and Environment, Prince of Songkla University, Phuket, Thailand; 2 Coastal Oceanography and Climate Change Research Center, Prince of Songkla University, Songkhla, Thailand; 3 Behavioural Ecology Laboratory, Department of Biology, Faculty of Science, Chulalongkorn University, Bangkok, Thailand; 4 Center of Excellence in Integrative Insect Ecology, Department of Biology, Faculty of Science, Chulalongkorn University, Bangkok, Thailand

**Keywords:** Nudibranch, Neuston, Andaman Sea, Bacteria, 16S rRNA gene amplicon

## Abstract

Sampling neustonic nudibranchs is challenging because they live in the open-ocean surface layer, which restricts available microbiome reference data. On July 12, 2025, a mass stranding of the blue dragon nudibranch (*Glaucilla marginata*) at Karon Beach, Phuket, Thailand, provided a rare opportunity for microbiome sampling. We generated a 16S rRNA gene amplicon dataset from whole-body homogenates of stranded individuals. Ten specimens were sequenced as five pooled samples (two individuals per pool) targeting the V3–V4 region on an Illumina MiSeq. After processing, the dataset contained 43 amplicon sequence variants, dominated by a small number of taxa; most samples were enriched in Firmicutes, largely represented by *Mycoplasma*. This low bacterial richness suggests host-mediated filtering, potentially driven by host-produced antibacterial compounds or competitive exclusion mediated by resident microbes. In addition, six culturable bacterial isolates were obtained and identified by near full-length 16S rRNA gene sequencing. Some isolates were not detected in the amplicon data, underscoring the importance of integrating culture-dependent and -independent approaches to better characterize host-associated assemblages.

## Introduction

Marine animals harbor complex microbial communities with vital roles in host nutrition, development, defense, and resistance to pathogens. In aquatic animals, diet and habitat are major determinants of the symbiotic bacterial composition (Langenheder & Lindström, 2019). In marine systems, many studies on host–microbe dynamics in benthic and nearshore organisms, particularly corals, sponges, bivalves, and echinoderms, have been performed mostly due to the relative ease of sampling and the well-documented nature of the host–environment interactions (Thomas et al., 2016; Carrier & Reitzel, 2019; Van Oppen & Blackall, 2019; Gignoux-Wolfsohn et al., 2024). However, whether these patterns persist in neustonic nudibranchs remains unclear.

Nudibranchs are particularly compelling, as many species feed on chemically defended prey, such as sponges, hydroids, and bryozoans, and have evolved to sequester prey-derived compounds or structures for their own protection (Gris & Prinsep, 2026). Previous studies of nudibranch-associated microbiomes primarily focused on benthic, reef-associated species (*e.g.*, symbionts) (Ng et al., 2022; Mahmoud et al., 2023; Stuij et al., 2023; Gallo et al., 2025). In these systems, hosts maintain a core set of symbiotic bacteria alongside a more variable microbial component influenced by the surrounding environment, prey availability, and host condition. In contrast, the microbiomes of neustonic and pelagic invertebrates living at the air–sea interface remain poorly understood, despite their potential to experience distinct microbial exposures and dietary inputs compared to benthic taxa (Albuquerque et al., 2021; Helm, 2021).

The blue dragon sea slug, *Glaucilla marginata* Reinhardt & Bergh, 1864 (synonymised name *Glaucus marginatus*), is a neustonic nudibranch drifting on the surface in the Indo-Pacific and Indian Oceans (Churchill, Valdés & Foighil, 2014b; MolluscaBase, 2026). It is sporadically found when wind and wave conditions concentrate individuals along shorelines (Churchill, Valdés & Foighil, 2014a; Gosliner, Valdés & Behrens, 2018). Their pelagic, surface-drifting lifestyle differs significantly from most nudibranchs, which are benthic and commonly associated with reef systems or other submerged substrates (Gosliner, Valdés & Behrens, 2018). Although *G. marginata* is accessible through surface-collecting methods such as neuston net tows and hand collection (Churchill, Valdés & Foighil, 2014a), encounters are often opportunistic, which makes sampling challenging. As a result, fundamental biological data, particularly on host-associated microbiomes, remain sparse compared with those of reef-dwelling counterparts.

Blue dragons target floating hydrozoan prey and are known for sequestering undischarged nematocysts and incorporating them into their tissues as a defensive arsenal (Yamamoto et al., 2025). Such intimate prey interactions may serve as a vector for prey-associated bacteria, potentially selecting specialized microbial lineages adapted to the unique chemical landscape of both the prey’s defensive means and the host’s internal environment (Krueger, Shore & Reitzel, 2022).

Furthermore, baseline microbiome profiles for *G*. *marginata* from the Andaman Sea region are notably scarce. Thus, obtaining fundamental microbiome data for this species is a crucial step toward broader comparative studies across diverse geographic regions, seasons, and environmental conditions.

**Figure 1:**
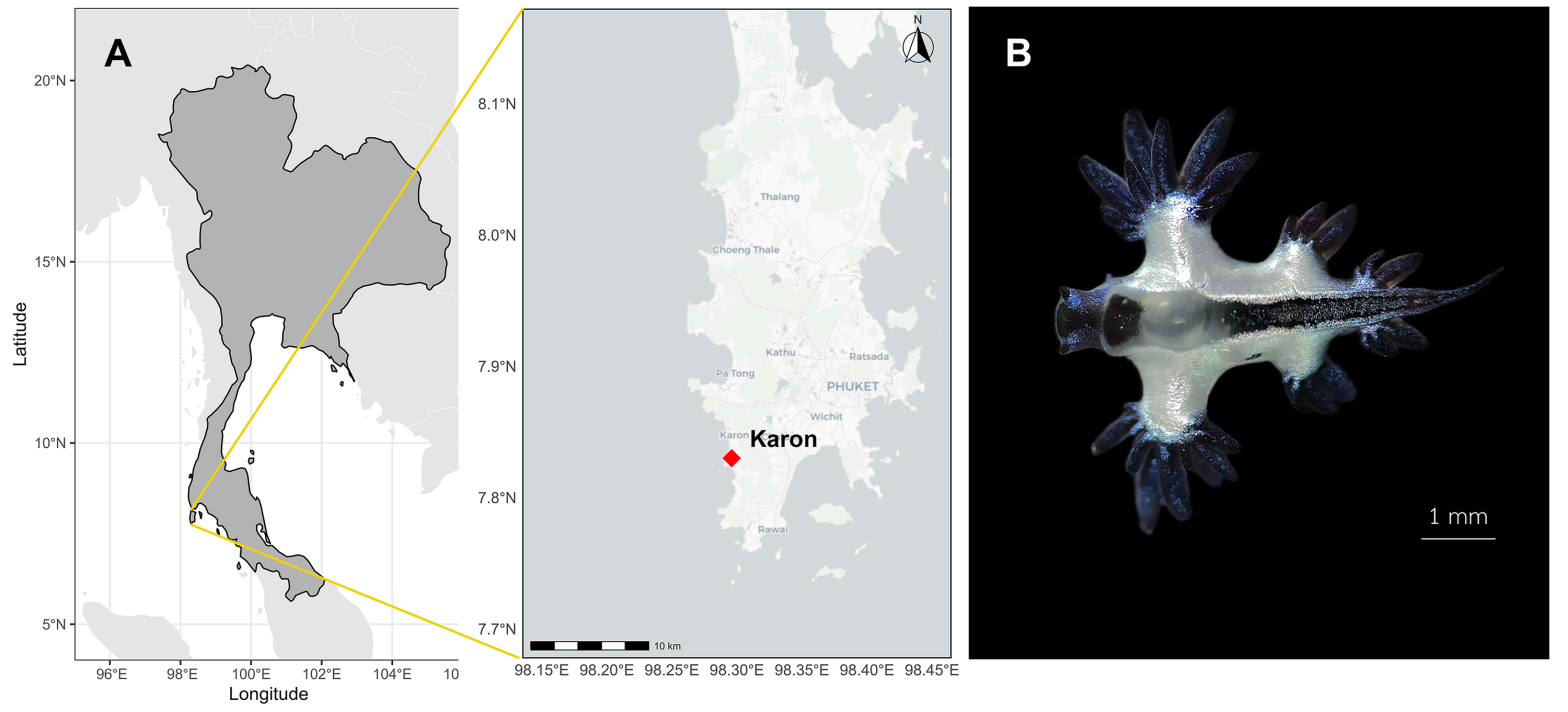
Sampling of blue dragon sea slug *Glaucilla marginata* specimens. (A) Sampling site at Karon Beach, Phuket, Thailand. The map was generated by the authors in R. The country-level boundary data for Thailand were obtained from Natural Earth, which provides public-domain map data. The detailed Phuket basemap uses OpenStreetMap data ©OpenStreetMap contributors, licensed under the Open Database License (ODbL). (B) Stereomicroscope image showing the ventral side of a representative individual. The photograph was taken by the authors.

On July 12, 2025, a massive stranding event of *G. marginata* along Karon Beach in Phuket, southern Thailand (Fig. 1A) provided a rare opportunity for systematic sampling of this neustonic species in the Andaman coastal region, where offshore collection is otherwise challenging. Here, we collected blue dragon sea slugs and characterized their associated bacterial communities using 16S rRNA gene amplicon sequencing. These data provide a baseline reference for future comparative research across locations, seasons, prey, and host conditions.

## Materials & Methods

### Sample collection and preparation

Live, freshly stranded blue dragon sea slugs *G. marginata* were collected along Karon Beach (7.836°N 98.294°E), Phuket, Thailand, on July 12, 2025 (Fig. 1A). Most specimens were found near the swash line often alongside by-the-wind sailors (*Velella velella* (Linnaeus, 1758)) and blue buttons (*Porpita porpita* (Linnaeus, 1758)). Individuals measuring 4–8 mm in length (Fig. 1B) were identified based on external morphology and geographic occurrence (Churchill, Valdés & Foighil, 2014b). To minimize shifts in bacterial community composition associated with postmortem decomposition, only intact, live individuals were sampled. Specimens were placed in a bottle containing natural seawater and transported to the laboratory within 1 h of collection.

In the lab, to minimize bacterial contamination from the surrounding environment, specimens were rinsed several times with sterile seawater and euthanized by freezing. Whole individuals were then homogenized in sterile microtubes using a glass rod; two individuals were pooled per sample to obtain sufficient DNA, yielding five pooled samples for downstream analyses. Homogenates were preserved in nucleic acid preservation buffer (NAPseq, BIOENTIST, Thailand) and stored at −20 °C until further processing.

### DNA extraction and sequencing

Metagenomic DNA was extracted using the DNeasy PowerSoil Pro DNA Kit (Qiagen, Hilden, Germany). The V3–V4 region of the 16S rRNA gene was amplified using overhang adapter-linked primers 341F (5′-CCTACGGGNGGCWGCAG-3′) and 805R (5′-GACTACHVGGGTATCTAATCC-3′) (Herlemann et al., 2011; Klindworth et al., 2013) with 2 × sparQ HiFi PCR Master Mix (QuantaBio, Beverly, MA, USA). Polymerase chain reaction (PCR) cycling consisted of initial denaturation at 98 °C for 2 min, followed by 28 cycles of 98 °C for 20 s, 60 °C for 30 s, and 72 °C for 30 s, and a final extension at 72 °C for 1 min. Amplicons were then purified using sparQ PureMag Beads (QuantaBio) and indexed using Nextera XT index primers (2.5 µL of each primer) in a 50 µL PCR reaction, followed by eight additional cycles under the same cycling conditions. Then, the indexed libraries were cleaned, pooled, and diluted to a final loading concentration of 6 pM. Cluster generation and sequencing were conducted on an Illumina MiSeq platform using 250-bp paired-end reads at the Omics Sciences and Bioinformatics Center, Chulalongkorn University (Bangkok, Thailand).

### Bioinformatics

Microbiome sequence processing and downstream analyses were conducted in QIIME 2 v2024.10 (Bolyen et al., 2019). Adapter trimming, denoising, error correction, paired-end read merging, and chimera removal were performed using DADA2 implemented in q2-dada2 (Callahan et al., 2016). The resulting amplicon sequence variants (ASVs) were used for downstream analyses. Features classified as mitochondria or chloroplasts were filtered out using the q2-taxa plugin before analysis. For phylogenetic diversity analyses, ASVs were inserted into the SILVA reference phylogeny using q2-SEPP (sepp-refs-silva-128.qza) (Janssen et al., 2018). This version was used because it is the optimized reference package provided for the q2-fragment-insertion plugin. Taxonomic classification was conducted separately using q2-feature-classifier with a naïve Bayes classifier trained on the SILVA 138 (99% OUTs) reference database (Bokulich et al., 2018). The use of different SILVA releases reflects the different requirements of the two analytical steps: phylogenetic insertion requires a compatible reference tree, whereas taxonomic classification requires a trained classifier for assigning ASV taxonomy. To standardize sequencing depth across samples, the feature table was rarefied to 24,957 sequences per sample (subsampling without replacement). Alpha diversity (measured by observed ASVs, Chao1 richness (Chao, 1984), Shannon diversity (log base 2) (Shannon, 1948), Simpson diversity (1 − D) (Simpson, 1949), and Pielou’s evenness (Pielou, 1966) were calculated in R v4.5.2 (R Core Team, 2025) using the vegan package (Oksanen et al., 2024) (Code S1). Bray-Curtis (Bray & Curtis, 1957) and weighted UniFrac (Lozupone et al., 2007) distance matrices were generated in QIIME 2 and imported into R v4.5.2 (R Core Team, 2025), where principal coordinates analysis (PCoA) (Gower, 1966) was performed (Codes S2 and S3). Shared and unique ASVs among the five pooled *Glaucilla marginata* samples were visualized using a Venn diagram generated in R with the VennDiagram package (Chen & Boutros, 2011). For each sample, ASV lists were prepared by removing missing values and duplicate ASV entries before plotting the overlap among samples (Code S4). For visualization of core ASVs, ASVs with a prevalence ≥80% were defined as core taxa. Their representative sequences were aligned in R using DECIPHER (AlignSeqs) (Wright, 2016), and a neighbor-joining tree based on maximum-likelihood distances was inferred using phangorn (Schliep, 2011), midpoint-rooted, and visualized as a circular phylogram with ggtree (Yu et al., 2017) and an abundance heatmap (Code S5).

### Bacterial isolation

Blue dragon sea slugs were processed for bacterial isolation in parallel with DNA extraction. Five individuals were pooled and homogenized in sterile seawater before spreading the homogenate onto Marine Agar 2216 (HiMedia, Maharashtra, India). After incubating the plates at room temperature for 48 h, colonies with distinct morphologies were selected and repeatedly restreaked on Marine Agar 2216 until pure cultures were obtained.

### Identification of bacterial isolates and comparison with amplicon-derived ASVs

Genomic DNA was extracted from bacterial isolates using a boiling lysis method with InstaGene™ Matrix; the crude lysate was used as PCR template. Nearly full-length 16S rRNA gene fragments were amplified using universal bacterial primers 27F (5′-AGAGTTTGATCMTGGCTCAG-3′) and 1492R (5′-TACGGYTACCTTGTTACGACTT-3′) (Weisburg et al., 1991) in 20 µL reactions containing 10 µL Axen™ H Taq PCR Master Mix (2 ×), 0.5 µL each for forward and reverse primers (10 pmol/µL; total 1 µL), 1–2 µL template DNA, and nuclease-free water to a final volume of 20 µL. PCR was performed under the following cycling conditions: initial denaturation at 95 °C for 5 min; 31 cycles of denaturation at 95 °C for 30 s, annealing at 57 °C for 30 s, and extension at 72 °C for 1 min 24 s; followed by a final extension at 72 ° C for 10 min and a hold at 4 °C. Amplicons were then subjected to bidirectional Sanger sequencing using the BigDye Terminator Cycle Sequencing Kit v3.1 on an ABI PRISM 3730XL DNA Analyzer (96-capillary), using internal primers 785F (5′-GGATTAGATACCCTGGTA-3′) and 907R (5′-CCGTCAATTCMTTTRAGTTT-3′) for sequencing of the 16S rRNA gene by Macrogen (Korea). The obtained 16S rRNA gene sequence was compared with that of the type strain from the EzBioCloud database (Yoon et al., 2017). A 16S rRNA sequence similarity threshold of 98.7% was used as a criterion for species-level assignment, as suggested by Chun et al. (2018).

In addition, to determine if the cultured isolates were present in the amplicon dataset, the nearly full-length 16S rRNA sequences of the bacterial isolates were compared against the ASV representative sequences using a local BLASTn search (BLAST+ v2.17.0; accessed 30 April 2026) (Camacho et al., 2009) across the V3–V4 region.

## Results

### Amplicon sequencing results and alpha diversity

**Figure 2:**
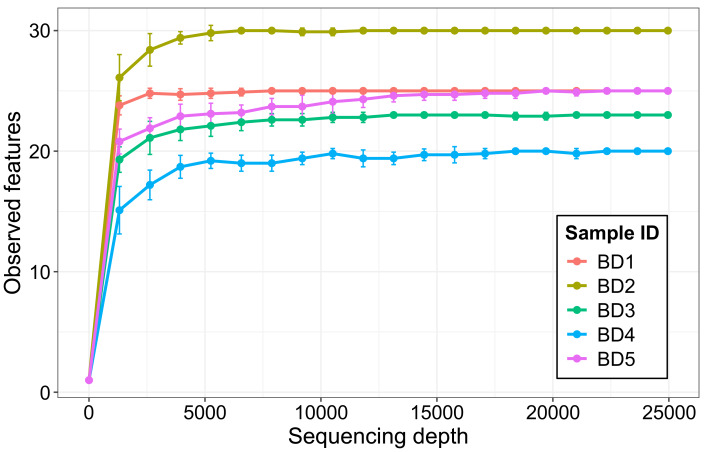
Rarefaction curve between observed ASVs and sequencing depth of bacterial communities from blue dragon sea slug samples. A higher number of observed ASVs indicates greater bacterial richness, whereas curves approaching a plateau suggest that sequencing depth was sufficient to capture most detectable bacterial diversity in the samples.

Microbial profiling of *G. marginata* was conducted using 16S rRNA gene (V3–V4) amplicon sequencing from whole-body tissue. Illumina sequencing generated 336,485 reads from five pooled samples. Raw sequencing reads have been deposited in the NCBI Sequence Read Archive (SRA) under BioProject PRJNA1416254 (SRA accessions SRR37058219 –SRR37058223). After filtering to remove low-quality and chimeric reads, 199,719 reads were subjected to further processing (Table S1). To account for differences in sequencing depth, all samples were rarefied to 24,957 reads before taxonomic assignment (Fig. 2). A total of 43 ASVs were identified across samples (Table S2).

Bacterial richness and alpha diversity were broadly similar across samples. The observed ASV counts were identical to Chao1 estimates for every sample, ranging from 20 to 30 (Table 1). Mean Shannon and Simpson diversity indices were 3.06 and 0.83, respectively and Pielou’s evenness was moderate, 0.66 on average.

**Table 1:** Bacterial richness and alpha-diversity indices of bacterial communities in blue dragon sea slug samples. Observed ASVs and Chao1 represent estimates of bacterial richness, with higher values indicating a greater number of detected or estimated bacterial taxa. Shannon diversity reflects both richness and evenness, whereas Simpson diversity (1−D) gives greater weight to dominant taxa. Evenness indicates how evenly ASV abundances are distributed within each sample, with higher values reflecting a more even community.

**Sample**	**Observed ASVs**	**Chao1**	**Shannon**	**Simpson (** **1−D)**	**Evenness**
BD1	25	25	3.27	0.86	0.71
BD2	30	30	3.16	0.84	0.64
BD3	23	23	2.91	0.80	0.64
BD4	20	20	2.60	0.77	0.60
BD5	25	25	3.35	0.88	0.72
Mean	24.60	24.60	3.06	0.83	0.66
SD	3.65	3.65	0.31	0.05	0.05

### Beta diversity

**Figure 3:**
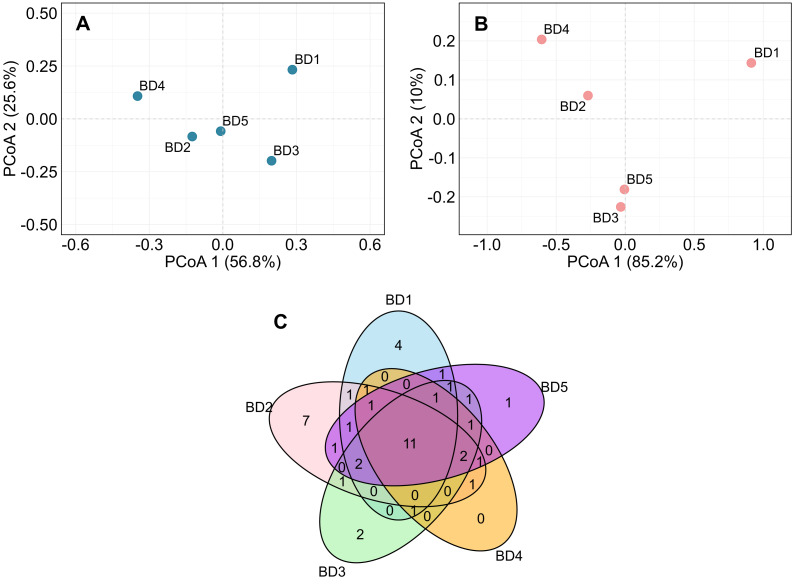
Beta-diversity patterns and shared amplicon sequence variants (ASVs) among blue dragon sea slug samples. Principal coordinates analysis (PCoA) ordinations based on (A) Bray-Curtis dissimilarity and (B) weighted UniFrac distance show variation in bacterial community composition among the five pooled *G. marginata* samples. Bray-Curtis dissimilarity reflects differences in ASV abundance composition, whereas weighted UniFrac incorporates both relative abundance and phylogenetic relatedness among ASVs. Greater separation between samples indicates greater dissimilarity in bacterial community structure. (C) Venn diagram showing the number of shared and unique ASVs among samples BD1–BD5, highlighting both the conserved and sample-specific components of the blue dragon sea slug microbiome.

PCoA ordinations showed differences in bacterial community composition among the five *G. marginata* samples (Figs. 3A, 3B). Based on Bray-Curtis dissimilarity, the mean pairwise dissimilarity among samples was 0.464 ± 0.108 (Table S3). Samples were separated primarily along PCoA1 (56.8% of variation) and PCoA2 (25.6%), with one sample (BD1) separate from the remaining samples, indicating a distinct community composition. Based on weighted UniFrac distance, the mean pairwise distance among samples was 0.777 ± 0.401 (Table S4). Weighted UniFrac distance revealed an even stronger separation along PCoA1 (85.2% of variation), placing BD1 far from the other samples and indicating pronounced phylogenetic differences in community structure; in contrast, BD3 and BD5 clustered most closely under weighted UniFrac. Despite this sample variation, 11 ASVs were shared by all samples (Fig. 3C, Table S2). These shared ASVs were dominated by members of Flavobacteriaceae, unclassified Gammaproteobacteria, unclassified Nitrincolaceae, and *Endozoicomonas*. Each sea slug sample also contained a small number of unique ASVs, indicating the presence of both shared and sample-specific bacterial components.

### Community composition

**Figure 4:**
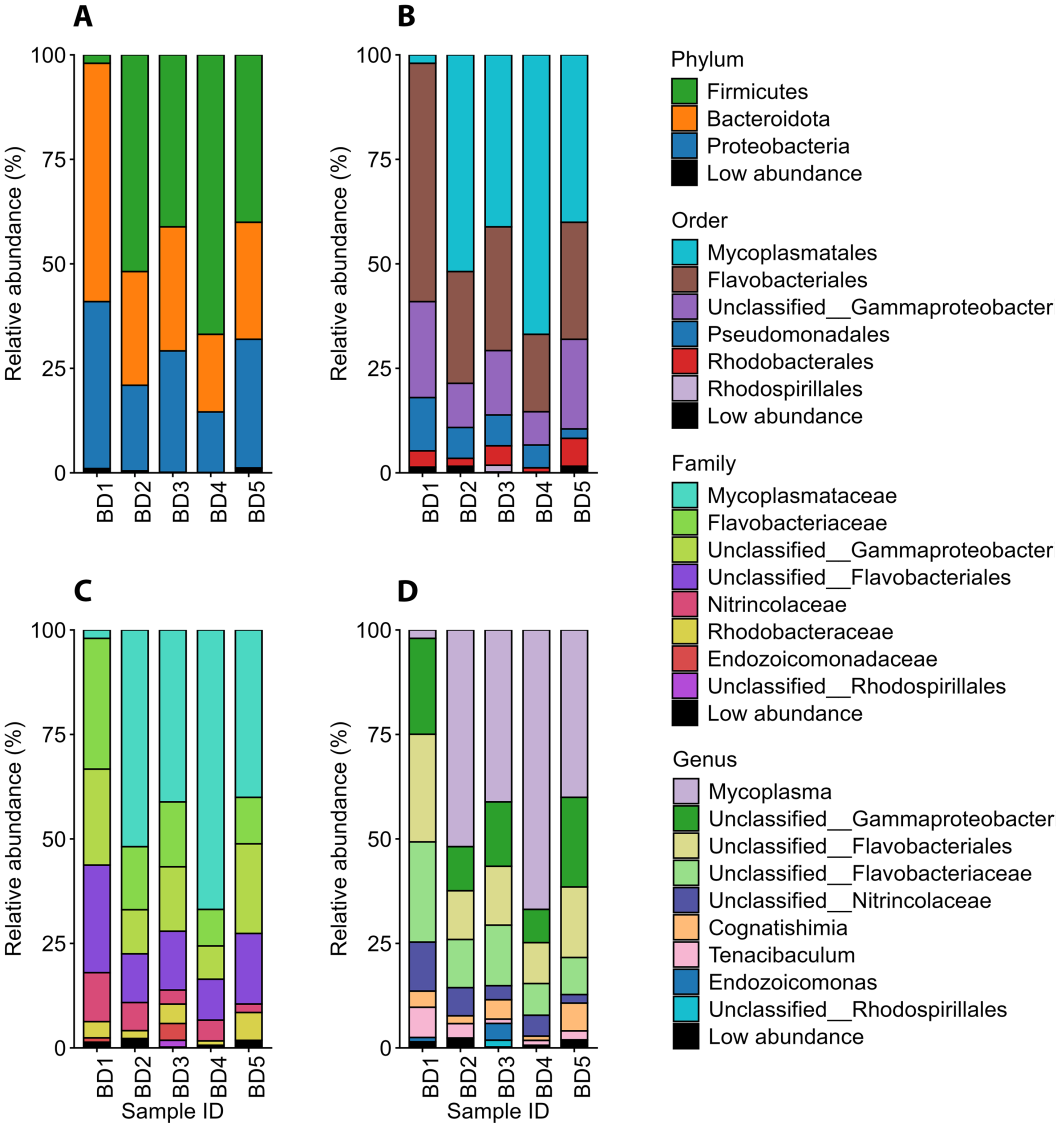
Relative abundance of bacterial taxa in blue dragon sea slugs. (A) Phylum level, (B) order level, (C) family level, and (D) genus level. Taxa with relative abundance <1% were grouped as low-abundance taxa.

Overall, Firmicutes was the dominant phylum, exclusively represented by *Mycoplasma* (Fig. 4). The next most abundant phyla, Bacteroidota and Proteobacteria, were taxonomically more diverse. Within Bacteroidota, most reads were assigned to unclassified *Flavobacteriaceae*, followed by *Tenacibaculum*; *Prevotellaceae* and *Chitinophagaceae* were detected at low abundance (<1%; Table S5). Proteobacteria were dominated by Gammaproteobacteria, although only a small proportion of ASVs could be resolved up to the genus level (*e.g.*, *Cognatishimia*, *Endozoicomonas*, *Methylibium*, and *Chujaibacter*). Other phyla, including Verrucomicrobiota, Patescibacteria, Actinobacteriota, and Chloroflexi, each contributed <1% relative abundance.

**Figure 5:**
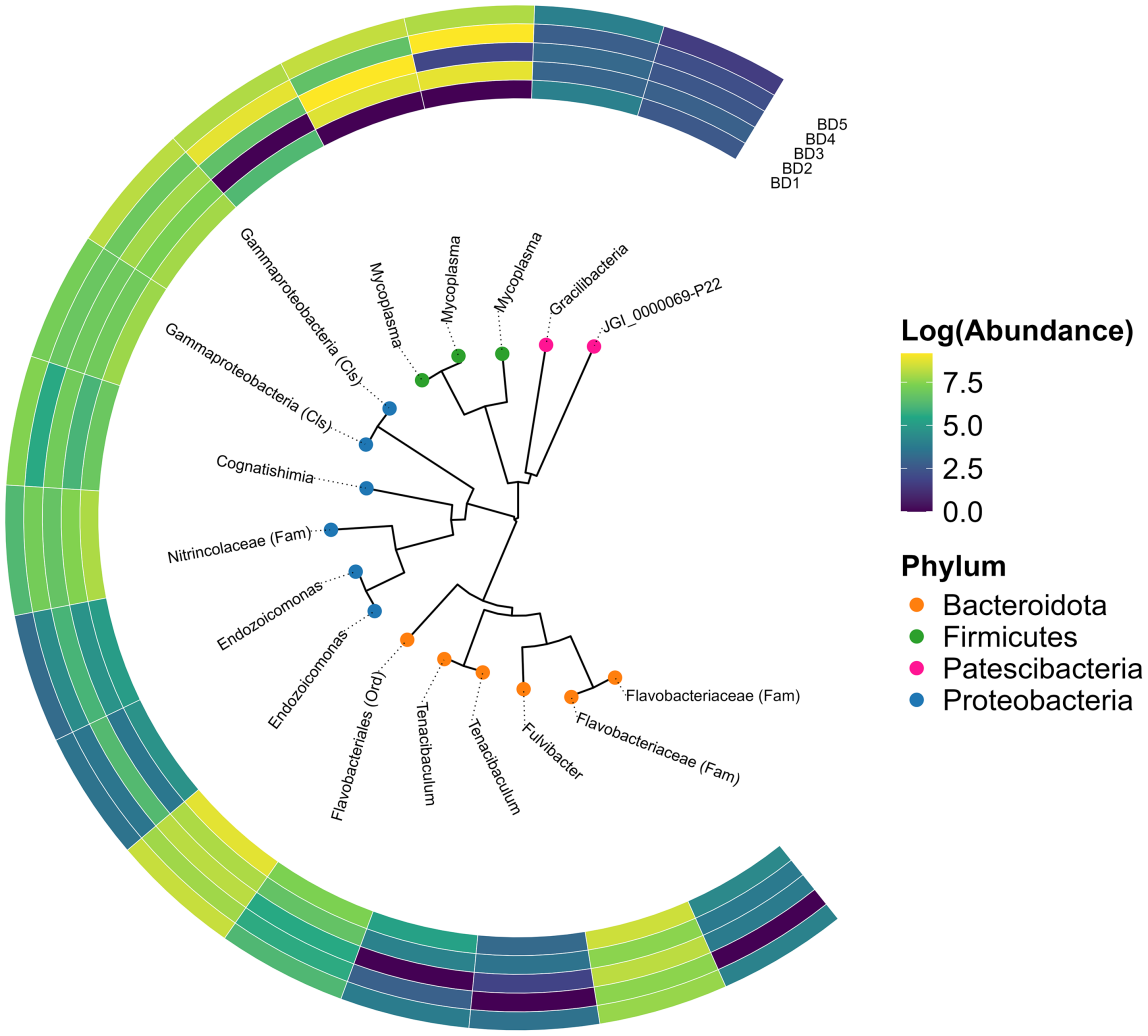
Phylogenetic tree of core ASVs (prevalence >  80% across samples) showing their log-transformed relative abundance across five blue dragon sea slug samples (BD1–BD5).

Phylogenetic analysis of core ASVs (prevalence ≥80% across samples, Table S6) showed clustering into four phyla—Firmicutes, Proteobacteria, Bacteroidota, and a minor Patescibacteria clade (Fig. 5). Core Firmicutes ASVs formed a distinct *Mycoplasma* cluster that remained consistently high in log abundance across samples, although individual ASVs differed in their relative contributions, indicating a stable dominant lineage. In contrast, core Bacteroidota ASVs (Flavobacteriaceae-related taxa and *Tenacibaculum*) exhibited greater interindividual variability, suggesting sample-specific enrichment. Similarly, core Proteobacteria ASVs, mainly affiliated with Gammaproteobacteria, were widespread, generally occurring at moderate-to-high abundance. Although Patescibacteria accounted for <1% of total reads, it was consistently present, thus satisfying the core criterion.

### Bacterial isolates from blue dragon sea slugs

Blue dragon sea slug–associated bacteria were successfully isolated on Marine Agar 2216. Based on 16S rRNA gene sequencing, six isolates were identified: two *Photobacterium lucens*, two *Endozoicomonas atrinae*, one *Vibrio harveyi*, and one *V. campbellii* (Table 2). Their sequences have been deposited in GenBank under accession numbers PX915876 –PX915881. A direct sequence comparison revealed that both *E. atrinae* isolates (PSUxCU-BD6 and PSUxCU-BD8) shared 100% sequence identity with ASVs from the metabarcoding dataset (Table S7). Additionally, *V. harveyi* (PSUxCU-BD5) and *V. campbellii* (PSUxCU-BD7) demonstrated >99% identity to ASVs (99.5% and 99.1%, respectively). Conversely, the two *P. lucens* isolates were not recovered in the amplicon dataset, with their highest sequence identity to any ASV reaching only 85.9%.

**Table 2:** Bacteria isolated from blue dragon sea slug samples. Bacterial isolates were identified by near full-length 16S rRNA gene sequencing. Taxonomic identities were assigned based on sequence similarity to type-strain reference sequences in the EzBioCloud database, with corresponding GenBank accession numbers provided for the closest matches.

**Isolate ID** **(GenBank accession number)**	**Closest species** **(GenBank accession number)**	**Similarity (%)**
PSUxCU-BD1 (PX915876)	*Photobacterium lucens* (SSHW01000013)	99.69
PSUxCU-BD2 (PX915877)	*Photobacterium lucens* (SSHW01000013)	99.08
PSUxCU-BD5 (PX915878)	*Vibrio harveyi* (BCUF01000119)	99.59
PSUxCU-BD6 (PX915879)	*Endozoicomonas atrinae* (KC878324)	99.73
PSUxCU-BD7 (PX915880)	*Vibrio campbellii* (AMDG01000189)	99.79
PSUxCU-BD8 (PX915881)	*Endozoicomonas atrinae* (KC878324)	99.18

**Notes.**

Isolates PSUxCU-BD3 and PSUxCU-BD4 were lost during subculturing and were not viable for molecular identification.

## Discussion

In this study, we obtained baseline microbiome data from *G. marginata* by combining 16S rRNA gene (V3–V4) amplicon profiles from whole-body samples with culture-based isolation and near full-length 16S rRNA gene identification. Together, these datasets offer an initial microbiome “snapshot” for a neustonic nudibranch in the Andaman Sea region and a reference for future comparisons across neustonic invertebrate and sea slug microbiomes. Across the five pooled samples, we detected 43 ASVs in total (20–30 ASVs per sample). The equality between observed ASV richness and Chao1 estimates within each sample suggest minimal unobserved richness at the sequencing depth used.

Overall, the blue dragon sea slug microbiome showed comparatively low richness and a taxonomic profile different from that of many reef-dwelling, sponge-feeding nudibranchs, which often exhibit higher alpha diversity and are typically dominated by Proteobacteria (Mahmoud et al., 2023; Stuij et al., 2023; Gallo et al., 2025). Similarly, Ng et al. (2022) reported low richness in the reef-associated nudibranch *Pteraeolidia semperi*—a hydroid-feeding species that harbors symbiotic zooxanthellae (Soon et al., 2023)—and Firmicutes enrichment largely driven by *Mycoplasma*. Overall, our dataset suggests the dominance of Firmicutes/*Mycoplasm* a communities in nudibranchs with trophic strategies as well as symbiotic associations are distinct from sponge-feeding taxa (Ng et al., 2022).

Against the typical seawater background—with generally diverse prokaryotic communities in Indian Ocean waters, often dominated by Proteobacteria and Cyanobacteria (Jasna, Nathan & Parvathi, 2020; Prasoodanan et al., 2024)—the comparatively low richness observed here, together with the high relative abundance of *Mycoplasma* and Flavobacteriales, likely reflects host-mediated filtering rather than passive acquisition from the environment.

Although the mechanisms underlying this filtering remain unclear, several explanations are plausible. One is that the host produces antimicrobial compounds that constrain colonization. Antimicrobial activity has been reported from tissue extracts of several sea slugs; in some cases, these bioactivities could derive from dietary metabolites (Gunthorpe & Cameron, 1987; Uddin et al., 2009; Gris & Prinsep, 2026). In addition, blue dragon sea slugs are well known for accumulating nematocysts from cnidarian prey (Yamamoto et al., 2025; Gris & Prinsep, 2026). Such host- or diet-associated compounds could suppress many bacteria, allowing only a limited set of tolerant or host-adapted lineages to dominate.

The beta-diversity patterns observed here indicate considerable microbiome variability among stranded individuals, despite the overall low richness of the bacterial communities. The stronger separation observed in weighted UniFrac than in Bray-Curtis space suggests that the observed dispersion was driven not only by shifts in relative abundance, but also by differences in phylogenetic composition. Such heterogeneity is plausible for stranded individuals and may reflect variations in recent diet, host age and reproductive stage, microhabitat exposure before stranding, and the acquisition of transient or opportunistic taxa from either the water column or prey (Shade et al., 2013; Langenheder & Lindström, 2019). Nonetheless, the persistence of repeatedly detected taxa across samples (*e.g.*, *Mycoplasma*, unclassified Flavobacteriaceae, and unclassified Gammaproteobacteria) is consistent with a microbiome containing a conserved “core” alongside a more flexible, sample-specific component.

Another possibility to explain the low diversity and variation of ASV between individuals could be competitive exclusion mediated by resident microbes, which could further reinforce low diversity. Many marine-invertebrate-associated bacteria—including *Vibrio*, *Pseudoalteromonas*, *Marinomonas*, and *Nocardiopsis*—can produce antibacterial compounds that inhibit competitors, thus shaping community composition (Böhringer et al., 2017; Kristiana et al., 2020; Abdelrahman et al., 2021; Sabdono et al., 2022). In blue dragon sea slugs, antagonistic interactions could restrict colonization to taxa with sufficient antimicrobial tolerance. *Mycoplasma* dominates in this context because this species naturally lacks a cell wall, making it resistant to antimicrobials that target cell walls (Gautier-Bouchardon, 2018). These considerations support the idea that blue dragon sea slugs may represent a chemically and biologically selective niche where diet-derived toxins, host defenses, and microbial antagonism limit bacterial richness, promoting dominance by a few lineages, such as *Mycoplasma* and Flavobacteriales.

However, the lack of key comparative datasets (*e.g.*, co-occurring seawater, prey items, or other nudibranch species) prevents us from determining whether the observed dispersion is typical for *G. marginata* or the differences between these communities and the environment. In particular, without profiling prey microbiomes, we cannot assess the extent to which host-associated taxa may be diet-derived. Despite limited microbiome data for hydrozoans—the principal prey of blue dragon sea slugs—studies on related gelatinous zooplankton have reported *Mycoplasma* enrichment in true jellyfish such as *Aurelia aurita* and *Rhizostoma pulmo* (Weiland-Bräuer et al., 2015; Stabili et al., 2020), raising the possibility that *Mycoplasma* may be associated with gelatinous prey or prey-linked ecological niches. Targeted comparisons among host, prey, and surrounding seawater, coupled with antimicrobial assays of host tissues and prey-derived extracts, are warranted to test these hypotheses.

Culture-based isolation provided complementary evidence not fully captured by amplicon sequencing alone. We isolated and identified six bacterial strains. Four of these (two isolates of *E. atrinae*, one of *V. harveyi*, and one of *V. campbellii*) shared >99% sequence identity with ASVs that were present at low relative abundance in the amplicon dataset. In contrast, the two *P. lucens* isolates were not detected in the amplicon data. The omission of some taxa is a recognized limitation of 16S rRNA gene sequencing. This discrepancy is typically attributed to primer bias, where universal primers fail to anneal to certain templates (Abellan-Schneyder et al., 2021), or insufficient sequencing depth, which may fail to capture rare taxa (Gweon et al., 2019). Consequently, this mismatch underscores the vital importance of integrating culture-dependent and culture-independent approaches to accurately characterize host-associated assemblages.

Ecologically, the culturable bacteria recovered in this study are highly relevant to the marine environment. *Vibrio* and *Photobacterium* are common marine taxa both free-living and associated with diverse marine animals, whereas *Endozoicomonas* is frequently reported in marine invertebrates, including many nudibranchs (Hyun et al., 2014; Ng et al., 2022; Stuij et al., 2023; Da Silva, Costa & Keller-Costa, 2025; Gallo et al., 2025). Note that our isolation protocol used marine agar, which selects for fast-growing heterotrophs, so additional culturable bacteria may have been missed.

## Conclusions

This study provides baseline microbiome data for the neustonic blue dragon sea slug *G. marginata* by combining 16S rRNA gene (V3–V4) amplicon sequencing of five pooled, whole-body samples with culture-based isolation. Across pools, we detected 43 ASVs (20–30 per pool) and observed broadly similar alpha-diversity patterns, indicating comparatively low bacterial richness. Communities were dominated by Firmicutes, represented exclusively by *Mycoplasma*, with recurrent taxa including unclassified Flavobacteriaceae (including *Tenacibaculum*) and Gammaproteobacteria, consistent with a conserved core and a variable component. Cultivation recovered six bacterial strains representing four species (*P. lucens*, *E. atrinae*, *V. harveyi*, and *V. campbellii*). This finding underscores the value of integrating cultivation with marker-gene profiling to capture host-associated taxa, including taxa that occur at low relative abundance or are not resolved in 16S amplicon data. Interpretation is limited by using pooled, whole-body homogenates from stranded individuals and the absence of paired seawater and prey microbiomes. Therefore, future work should aim to replicate our results across stranding events, seasons, and host conditions, incorporate matched host–prey–environment sampling, and use shotgun metagenomics and/or isolate genome sequencing to better resolve functional potential. Targeted assays could test whether antimicrobial activity contributes to the low richness and pronounced dominance observed here.

## Supplemental Information

10.7717/peerj.21462/supp-1 Supplemental Information 1 Bioinformatic data of bacterial communities associated with the blue dragon sea slug (*Glaucilla marginata*) samples Supplemental Tables

10.7717/peerj.21462/supp-2 Supplemental Information 2 R script for alpha diversity calculation The script imports the feature table, extracts sample count data, transposes the matrix so that samples are rows and ASVs are columns, and calculates observed richness, Chao1, ACE, Shannon diversity, Simpson diversity (1 − D), inverse Simpson diversity, and Pielou’s evenness using the vegan package in R. The output is exported as a CSV file.

10.7717/peerj.21462/supp-3 Supplemental Information 3 R script for principal coordinates analysis (PCoA) based on Bray-Curtis dissimilarity The script imports a Bray-Curtis distance matrix from a CSV file, performs ordination using classical multidimensional scaling via the cmdscale function, extracts the first two principal coordinate axes, and calculates the percentage of variance explained.

10.7717/peerj.21462/supp-4 Supplemental Information 4 R script for principal coordinates analysis (PCoA) based on weighted UniFrac distance The script imports a weighted UniFrac distance matrix from a CSV file, performs ordination using classical multidimensional scaling via the cmdscale function, extracts the first two principal coordinate axes, calculates the percentage of variance explained, and produces a publication-quality TIFF figure with sample labels using ggplot2 and ggrepel.

10.7717/peerj.21462/supp-5 Supplemental Information 5 R script for Venn diagram analysis of shared and unique ASVs among blue dragon sea slug samples This script is used to generate a Venn diagram showing shared and unique amplicon sequence variants (ASVs) among five pooled Glaucilla marginata samples. The script imports a CSV file containing ASV identifiers for samples BD1–BD5, removes missing values and duplicated ASV entries within each sample, creates sample-specific ASV lists, and generates a publication-quality Venn diagram using the VennDiagram package in R.

10.7717/peerj.21462/supp-6 Supplemental Information 6 R script for phylogenetic tree construction and abundance heatmap visualization of the top 50 ASVs The script imports an ASV feature table and representative DNA sequences, parses taxonomy annotations, selects the top 50 ASVs based on total abundance, aligns the corresponding sequences using DECIPHER, constructs a neighbor-joining phylogenetic tree using phangorn, roots the tree at the midpoint, and visualizes the resulting tree using ggtree. Tip labels are assigned based on the best available taxonomy level, and a log-transformed abundance heatmap is added as an outer ring.

10.7717/peerj.21462/supp-7 Supplemental Information 7 PCR Primers used for 16S rRNA gene amplicon sequencing and bacterial isolate identification PCR primers used in this study, including primers for amplification of the V3–V4 region of the bacterial 16S rRNA gene, commercial Nextera XT index primers used for Illumina library indexing, primers used for near-full-length 16S rRNA gene amplification of bacterial isolates, and internal primers used for Sanger sequencing.

## Additional Information and Declarations

### Data Availability

The following information was supplied regarding data availability: The data and code are available in the Supplemental Files. Raw sequencing reads are available in the NCBI Sequence Read Archive (SRA) under BioProject PRJNA1416254. 16S rRNA gene sequences of bacterial isolates are deposited in GenBank under accession numbers PX915876 –PX915881.
